# An Antioxidant Enzyme Therapeutic for Sepsis

**DOI:** 10.3389/fbioe.2021.800684

**Published:** 2021-11-23

**Authors:** Feifei Li, Ran Yan, Jun Wu, Zeren Han, Meng Qin, Chaoyong Liu, Yunfeng Lu

**Affiliations:** ^1^ Beijing Advanced Innovation Center for Soft Matter Science and Engineering, Beijing University of Chemical Technology, Beijing, China; ^2^ College of Life Science and Technology, Beijing University of Chemical Technology, Beijing, China; ^3^ Department of Chemical and Biomolecular Engineering, The University of California, Los Angeles, CA, United States

**Keywords:** sepsis, reactive oxygen species, cytokines, enzyme therepeutic, catalase

## Abstract

Sepsis is a systemic inflammatory response syndrome caused by infections that may lead to organ dysfunction with high mortality. With the rapid increase in the aging population and antimicrobial resistance, developing therapeutics for the treatment of sepsis has been an unmet medical need. Excessive production of reactive oxygen species (ROS) during inflammation is associated with the occurrence of sepsis. We report herein a treatment for sepsis based on PEGylated catalase, which can effectively break down hydrogen peroxide, a key component of ROS that is chemically stable and able to diffuse around the tissues and form downstream ROS. PEGylated catalase can effectively regulate the cytokine production by activated leukocytes, suppress the elevated level of AST, ALT, TNF-α, and IL-6 in mice with induced sepsis, and significantly improve the survival rate.

## Introduction

Sepsis is a life-threatening organ dysfunction caused by the host’s unbalanced response to infection. Septic shock is a type of sepsis in which the changes in metabolism, cells, and hemodynamics significantly increase the likelihood of fatality (Singer et al., 2016; Rhodes et al., 2017; Korneev, 2019). Relevant studies have shown that there are more than 19 million sepsis patients worldwide each year, of which 6 million patients die, and the case fatality rate is greater than 25%. About 3 millions of those who survived had cognitive impairments that severely affected their quality of life (Reinhart et al., 2017; Aziz and Wang, 2019). Septic shock is also one of the common clinical manifestations of severe patients with COVID-19 (Huang et al., 2020). People over 65 years of age, infants, immunocompromised patients, and patients with autoimmune diseases, tumors, kidney diseases, and lung diseases are the most susceptible to sepsis (Gotts and Matthay, 2016). At present, treatments for sepsis mainly include fluid therapy (crystal fluid and albumin), antibacterial drugs, vasoactive drugs (norepinephrine), glucocorticoids, injection immunoglobulin, etc (Rhodes et al., 2017). Due to factors such as individual difference, aging, and antimicrobial resistance, the morbidity and mortality of sepsis remain high.

It has been documented that cytokines (Rittirsch et al., 2008) and reactive oxygen species (ROS) (Mittal et al., 2014) play essential roles in sepsis. Reactive oxygen species (ROS) mainly come from cell respiration, protein folding, or various by-products of metabolism (Reczek and Chandel, 2015). In another source, it is mainly produced by NADPH oxidase, which mainly exists in phagocytes and vascular endothelial cells (Lambeth, 2004; Mittal et al., 2014). ROS mainly include superoxide anion (·O_2_^−^), hydroxyl radical (OH^·^), hydrogen peroxide (H_2_O_2_), and hypochlorous acid (HOCl), etc (Thannickal and Fanburg, 2000; Mittal et al., 2014; Reczek and Chandel, 2015). Partial reduction of O_2_ and electron-transfer reactions in the mitochondria generate O_2_^−^, which is converted to hydrogen peroxide (H_2_O_2_) mediated by superoxide dismutase (SOD) (Lambeth, 2004; Phaniendra et al., 2015). H_2_O_2_ may be subsequently reacted generating OH through the Fenton’s reaction (Reyhani et al., 2019), HOCl through myeloperoxidase (MPO), H_2_O through glutathione/glutathione peroxidase (GSH/GPX), and H_2_O/O_2_ through catalase (CAT) (Patlevič et al., 2016). Under pathological conditions, an unbalance of the generation and elimination of ROS results in oxidative stress with excess ROS. Since H_2_O_2_ is chemically stable and able to diffuse through cells and tissues, it may accumulate locally or systematically (Majewska et al., 2004) and activate the inflammatory response (Naik and Dixit, 2011).

Upon the occurrence of infection, leukocytes are attracted to affected sites and release cytokines and ROS (Qin et al., 2020). An excessive level of ROS may damage the biological macromolecules such as DNA, proteins, and lipids, which may cause dysfunction of cells and tissues (Yang et al., 2019) and further exacerbate the immune response. Uncontrolled production of ROS and cytokines may eventually lead to excessive inflammatory response and cytokine storm. Therefore, eliminating the excessively produced H_2_O_2_ helps to reduce the oxidative stress and to regulate the expression of pro-inflammatory cytokines, which is beneficial for the treatment of sepsis.

Organisms can effectively regulate their H_2_O_2_ levels through efficient enzymatic reactions. Catalase is the most abundant antioxidant enzyme commonly found in the liver, erythrocytes, and alveolar epithelial cells, and is the most effective catalyst for the decomposition of H_2_O_2_(Nishikawa et al., 2009). Catalase has attracted much attention in maintaining normal physiological functions and relieving pathological processes. However, exogenous catalase generally exhibits poor *in vivo* stability and short plasma half-life (only 6–10 min) (Qi et al., 2021), which preclude its broad use as therapeutics. Conjugation therapeutic proteins with poly (ethylene glycol) (PEG) is the golden standard to improve their pharmacokinetics and immunogenicity, which has been approved by the Food and Drug Administration (Abuchowski et al., 1977; Kinstler et al., 2002; Basu et al., 2006). Herein, we explore the use of PEG-conjugated catalase as a therapeutic treatment for sepsis. Our results suggest that PEGylated catalase can effectively regulate cytokine production by activated leukocytes, suppress the elevated level of AST, ALT, TNF-α, and IL-6 in mice with induced sepsis, and significantly improve the survival rate of the mice.

## Materials and Methods

### Materials

All reagents were used as received unless otherwise specified. Catalase (CAT) from *Aspergillus niger.* was purchased from Sunson Industry Group (Beijing, China). Methoxy polyethylene glycol acetic acid N-succinimidyl ester, Mw 20,000 (mPEG-NHS 20000) was purchased from JenKem Technology (Beijing, China). Trypsin and the 2,7-Dichlorodihydrofluorescein diacetate (DCFH-DA) were purchased from Sigma Aldrich (St. Louis, MO). Sulfo-Cyanine7 NHS ester was purchased from Beijing Okeanos Technology Co., Ltd. (Beijing, China). Lipopolysaccharides (LPS) (*Escherichia coli* 055: B5), Cell Counting Kit-8 (CCK-8), Bicinchoninic Acid (BCA) Protein Assay Kit, Catalase Activity Assay Kit, Endotoxin Erasol and Hoechst were purchased from Beijing Solarbio Science and Technology Co., Ltd. (Beijing, China). Tachypiens Amebocyte Lysate was purchased Zhanjiang Bokang Marine Biology Co., Ltd. (Zhanjiang, China). D-galactosamine (D-GalN) was purchased from J&k Scientific (Beijing, China). Human pulmonary alveolar epithelial (HPAEpi) cells were purchased from BeNa Culture Collection (Beijing, China). Alanine Aminotransferase Kit (ALT) and Aspartate Aminotransferase Kit (AST) were purchased from Abcam (Cambridge, United Kingdom). Mouse TNF-α enzyme-linked immunosorbent assay (ELISA) kit and mouse IL-6 ELISA kit were purchased from PeproTech (Suzhou, China). TUNEL Apoptosis Detection Kit was purchased Boster Biological Technology Co., Ltd. (Wuhan, China). Zeba Spin 7 K MWCO Desalting Columns was purchased from Thermo Fisher Scientific, Inc. (Waltham, MA). Amicon ultra centrifugal filter was purchased from Merck Millipore (Burlington, MA). Roswell Park Memorial Institute 1,640 Medium (RPMI), fetal bovine serum (FBS), penicillin/streptomycin (P/S, 1%), trypsin-EDTA (0.25%) were purchased from Corning (Corning, NY).

### Instruments

The purity of catalase was examined by a size-exclusion column with a separation range of 10,000 Da–10,00,000 Da BioCore SEC-300) using a high-performance liquid chromatography (HPLC, 1,260 Infinity II system, Agilent) system. Transmission electron microscope (TEM, HT7700, Hitachi Ltd.) was employed to characterize the morphologies of CAT-PEG. Dynamic light scattering (DLS) measurements were performed on a Zetasizer Nano instrument (Malvern Instruments Ltd., United Kingdom) with a 10–mW helium–neon laser and thermoelectric temperature controller. Fluorescent intensity was measured with a Spectra Max M2 plate reader (Molecular Devices). The bioluminescent imaging of the mice was imaged with the IVIS Imaging System (IVIS Spectrum, PerkinElmer). H and E images were taken using inverted fluorescence microscope fluorescence microscope (BK-FL4, ChongQing Optec Instrument Co., Ltd.). TUNEL images were taken using a confocal laser scanning microscopy (Leica TCS SP8). The complete blood count and blood biochemical examination were performed using auto biochemistry analyzer (BS-420, Mindray) and auto hematology analyzer (RJ-0C107223, Mindray), respectively.

### Animals

Balb/c mice (6 weeks old; weight range, 18–22 g) were purchased from Beijing Huafukang Biotechnology Co., Ltd. All mice received human care in accordance with the guidelines of the local Institute of Health on the care and use of laboratory animals. The mice were socially housed under standardized conditions of light (12 h day/night rhythm), temperature (22°C) and humidity (55%), environmental enrichment, and had access to food and water ad libitum.

### Synthesis of Catalase-PEG

Catalase (CAT) was conjugated with methoxy polyethylene glycol acetic acid N-succinimidyl ester (mPEG-NHS) through the reaction of NHS ester with primary amine. Briefly, CAT was dialyzed against phosphate buffer (200 mM, pH 8.0) to remove any amine-reactive salt. After dialysis, the CAT solution was diluted to 0.5 mg/ml with phosphate buffer (200 mM, pH 8.0), followed by addition of mPEG-NHS solution (100 mg/ml in anhydrous DMSO) at a molar ratio of 500:1 (mPEG-NHS to CAT). The reaction was kept at room temperature for 2 h to achieve the conjugation. The unreacted mPEG-NHS was removed by an ultracentrifugal filter with a molecular weight cutoff of 100 kDa using 1× Phosphate-Buffered Saline (PBS, pH 7.4) as the washing buffer. The resulting PEGylated CAT (CAT-PEG) was then placed at 4°C for further use.

### Fluorescence Labeling of Catalase and Catalase-PEG

For the imaging purposes, CAT and CAT-PEG were fluorescently labeled with sulfo-Cyanine7 NHS ester (sulfo-Cy7-NHS), respectively. Briefly, 1 mg sulfo-Cy7-NHS was first dissolved in 200 µl anhydrous DMSO to make a stock solution (5 mg/ml). To achieve the labeling, sulfo-Cy7-NHS was added to CAT or CAT-PEG at a molar ratio of 5:1 (sulfo-Cy7-NHS to CAT), and the reaction was kept at room temperature for 2 h under dark. Finally, excess sulfo-Cy7-NHS was removed using a desalting column equilibrated with 1× PBS (PH = 7.4). The resulting sample was then placed at 4°C for further use.

### Preparation of Endotoxin-free Catalase and Catalase-PEG

The endotoxins in the CAT or CAT-PEG sample were removed using Endotoxin Erasol according to the manufacture’s protocol. Briefly, endotoxin erasol and the samples were mixed at a volume ratio of 10:1. The mixture was then placed on ice for 5 min and incubated at 37°C for 5min. Afterwards, the mixture was centrifuged at 12,000 rpm for 10 min at room temperature to collect the upper supernatant. This process was repeated for at least three times until the endotoxin level in the sample was below 5 EU/ml. The endotoxin level was determined using the tachypleus amebocyte lysate method according to the manufacture’s protocol. All the reagents and vials used during the preparation and detection process should be endotoxin-free.

### Characterization of Catalase and Catalase-PEG

Sodium dodecyl sulfate polyacrylamide gel electrophoresis (SDS-PAGE). SDS-PAGE was carried out in a 10% (w/v) polyacrylamide resolving gel. Briefly, 15 μl of CAT (0.5 mg/ml) or CAT-PEG (0.5 mg/ml) solution was mixed with 5× loading buffer (Solarbio) and incubated at 70°C for 15 min. Then, the samples were applied to 10% (w/v) sodium dodecyl sulfate polyacrylamide gel for electrophoresis at 120 V for 60 min. The gel was stained with Coomassie Brilliant Blue R-250 staining solution (Solarbio) for 1 h at room temperature, and then de-stained in the solution of 10% glacial acetic acid and 30% methanol overnight.

Transmission electron microscopy (TEM). TEM sample was prepared by dropping 10 μl of CAT-PEG (0.5 mg/ml) on a copper grid, followed by staining using 1% phosphotungstic acid solution for 2 min. The grid was then rinsed three times with deionized water and dried for further observation with an HT7700 field emission electron microscope operated at 100 kV.

Dynamic light scattering (DLS) measurement. DLS experiments were performed with a Zetasizer Nano instrument equipped with a 10-mW helium-neon laser and thermoelectric temperature controller. The measurements were taken at 25°C with a 90° scattering angle. The sizes and the standard derivations of CAT and CAT-PEG were obtained by averaging the values of at least three measurements. Zeta potentials of CAT and CAT-PEG were determined by photon correlation spectroscopy using a Zetasizer Nano instrument. The measurements were performed at 25°C with a detection angle of 90°, and the raw data were subsequently correlated to Z average mean size using a cumulative analysis by the Zetasizer software package.

High pressure liquid chromatography (HPLC) measurement. HPLC measurement was conducted using an Agilent 1,260 Infinity Ⅱ high-performance liquid chromatography packed with a BioCore™ SEC column (NanoChrom Analytical Technology Co., Ltd.). The flow rate of the mobile phase (phosphate buffer, 10 mM, pH 8.0) was set at 1 ml/min to establish a baseline, after which 50 µl of CAT (1 mg/ml) or CAT- PEG (1 mg/ml) was injected under a flow rate of mobile phase at 0.5 ml/min. The signals of CAT and CAT- PEG were monitored by a UV detector at the wavelength of 405 nm.

### Determination of the Catalase and Catalase-PEG Concentrations

The concentrations of CAT and CAT-PEG were determined by optical absorption measurements using an extinction coefficient of ε= 324,000 M^−1^ cm^−1^ at 405 nm (Samejima and Yang, 1963). The concentration of Cy7-labeled CAT or CAT-PEG was determined with the BCA protein assay. Briefly, the BCA working reagent were prepared by mixing 50 volumes of BCA reagent with 1 volume of Cu reagent (50:1) together. Standard curves of CAT were established using CAT with a series of concentrations (0.03125, 0.0625, 0.125, 0.25, 0.5, 1 mg/ml). This was achieved by repeating 2-fold dilutions of 1 mg/ml CAT solution with PBS. Then the standard sample and Cy7-labeled CAT were mixed with the BCA working reagent in a volume ratio of 1:10, respectively, and incubated at 37°C for 30 min. The absorbance at 562 nm was determined with a UV/Vis spectrophotometer (NanoDrop, Thermo Fisher Scientific). Concentration of the Cy7-labeled CAT or CAT-PEG was calculated by using its absorbance at 562 nm and the standard curve established at the same condition.

### Determination of the Catalase and Catalase-PEG Activities

The activities of CAT and CAT-PEG were tested using a catalase activity assay kit according to the manufacture’s protocol. Briefly, 1 ml of H_2_O_2_ solution (pH = 7.4, 0.1 M HEPES buffer, H_2_O_2_ concentration 0.03% w/v) and 35 µl of sample were added to a 1 ml quartz cuvette and mixed for 5s. Then, the absorbance at 240 nm was measured immediately (A1) and after 1 min (A2), respectively. The activity of catalase was calculated by the following equation: $\text{catalase activity }\left(\text{U/mL}\right)\;=\left[\Delta A\times Vtotal÷\left(\varepsilon \times d\right)\times 10^{6}\right]÷Vsample÷T=678\times \Delta A$ . Where ΔA=A1−A2 ; Vtotal stands for the total volume of the reaction system, 1.035 ml; ε stands for the molar absorptivity of H_2_O_2_, 43.6 L/mol/cm; d stands for the optical path of cuvette, 1cm; Vsample stands for the volume of the sample added, 0.035 ml; T stands for the reaction time, 1min.

### Determination of the Catalase and Catalase-PEG Stabilities

To measure the proteolytic stability of CAT or CAT-PEG, trypsin was mixed with CAT or CAT-PEG at a final trypsin concentration of 50 μg/ml and a final CAT concentration of 0.1 mg/ml. The mixture was incubated at 37°C for 4 h, followed by the enzyme activity test using a similar method as abovementioned. To measure the psychological stability of CAT-PEG, CAT-PEG was diluted with 1× PBS to a final concentration of 0.1 mg/ml, and further incubated at 37°C for 24, 48, 72, 96 and 120 h, respectively. The enzyme activity was then tested using a catalase activity assay kit as abovementioned.

### Cytotoxicity of Catalase-PEG

The cytotoxicity of CAT-PEG was evaluated by measuring the cell viability after the incubation of human pulmonary alveolar epithelial (HPAEpi) cells with different amount of CAT-PEG. Briefly, HPAEpi cells were seeded into a 96-well plate (1×10^4^ cells/well, 100 μl/well) and cultured in RPMI1640 supplemented with 10% FBS and 1% P/S for 24 h. The cells were then incubated with 20, 100, 500, or 1,000 μg/ml of CAT-PEG for 24 h, respectively. The cell viability was then assessed using CCK-8 Kit (Hao et al., 2021; Zheng et al., 2021), and measured the absorbance at 450 nm by a plate reader. The viability of untreated cells was used as 100% during the data analysis.

### The Ability of Catalase-PEG to Eliminate the Intracellular Reactive Oxygen Species

The ability of CAT-PEG to regulate the intracellular ROS level was investigated in the HPAEpi cells with a fluorescent probe for ROS (DCFH-DA). Briefly, HPAEpi cells were seeded in a glass-bottomed cell culture dish (1×10^5^ cells/dish) and cultured in RPMI1640 for 24 h. Then, HPAEpi cells were incubated with 8, 16, and 40 μg/ml of CAT-PEG for 12 h, respectively, followed by incubation with 10 µM DCFH-DA in the dark for 30 min at 37°C. Then the intracellular ROS of the HPAEpi cells was induced by incubating 1 mM H_2_O_2_ with the cells for 30 min at 37°C. The cell nuclei were stained with Hoechst and washed extensively with a fresh RPMI1640 medium for further observation using a confocal laser scanning microscopy (Leica TCS SP8).

To quantify the intracellular level of ROS, HPAEpi cells were seeded in 96-well plate (1×10^4^ cells/well, 100 μl/well) and cultured in RPMI1640 for 24 h. Then cells were incubated with 8, 16, and 40 μg/ml of CAT-PEG for 12 h, respectively, followed by incubation with 10 µM DCFH-DA in the dark for 30 min at 37°C. Then the intracellular ROS of the HPAEpi cells was induced by incubating 1 mM H_2_O_2_ with the cells for 30 min at 37°C. After being rinsed with cold PBS three times, the DCFH-DA fluorescence was measured using a plate reader (Ex. = 488 nm, Em. = 525 nm).

### The Ability of Catalase-PEG to Prevent Cell Injury

The ability of CAT-PEG to prevent cell injury was evaluated in a H_2_O_2_-induced cell injury model (Qin et al., 2020). Briefly, HPAEpi cells were seeded into a 96-well plate (1×10^4^ cells/well, 100 μl/well) and cultured in RPMI1640 for 24 h. Then, HPAEpi cells were incubated with 8, 16, and 40 μg/ml of CAT-PEG for 12 h, respectively, followed by addition of H_2_O_2_ at a final concentration of 500 µM. The cells were cultured for another 24 h for cell viability test. The cell viability was measured and analyzed as abovementioned.

### The Ability of Catalase-PEG to Regulate the Production of Inflammatory Factors

An *in-vitro* model by co-culture of leukocytes and HPAEpi cells was used to confirm the ability of CAT-PEG to regulate cytokine production as previously reported (Qin et al., 2020). Leukocytes were separated from whole blood from a donor according to the protocol provided by Thermo Fisher Scientific. The model was induced by addition of lipopolysaccharides (LPS) at a final concentration of 1 μg/ml to the co-cultured leukocytes and HPAEpi cells to activate the leukocytes. CAT-PEG was then added at a final concentration of 8, 16, and 40 μg/ml, respectively, and further incubated with the cells for 12 h. The concentrations of TNF-α and IL-6 in the media were measured with an enzyme-linked immunosorbent assay kit following the protocols provided. HPAEpi cells without any treatment, HPAEpi cells treated with 1 μg/ml LPS, and co-cultured leukocytes and HPAEpi cells without any treatment were used as controls.

### Pharmacokinetics of Catalase and Catalase-PEG

To evaluate the pharmacokinetics, BALB/c mice (8 w, 22 ± 2 g, n = 3) received 5 mg/kg CAT or CAT-PEG through tail-vein injection, and blood samples were collected at 0.1, 1, 2, 4, 6, 12, 24,36 and 48 h post-injection. The serum was separated from the whole blood by centrifugation at 4,000 *g* for 10 min, and the serum catalase activity was assessed by the catalase activity assay kit as abovementioned. To plot the pharmacokinetics curve, the activity of each serum sample was normalized to the activity of the serum collected from the mice treated with CAT-PEG at 0.1 h, which was recorded as 100%.

### Biodistribution of Catalase and Catalase-PEG

To assess the biodistribution of CAT-PEG, CAT and CAT-PEG labeled with sulfo-Cy7-NHS were used for intravenous injection, respectively. BALB/c mice (8 w, 22 ± 2 g) received 5 mg/kg of sulfo-Cy7-labeled CAT or sulfo-Cy7-labeled CAT-PEG through tail-vein injection and were euthanized 6, 12, 24, and 48 h post-injection. The organs of these animals (heart, liver, spleen, lung, and kidney) were collected and imaged with an *in vivo* imaging system (IVIS spectrum). The fluorescence intensity of region-of-interests (ROI) was inspected by Living Image software. Each sample has triplicates to generate statistical significance.

### *In vivo* Therapeutic Efficacy of Catalase-PEG in the Sepsis Model

The sepsis model was established as previously reported (Lehmann et al., 1987; Silverstein, 2004; Poli-de-Figueiredo et al., 2008; Maes et al., 2016). Briefly, 20 healthy mice were evenly divided into two groups. Each mouse was intravenously injected with LPS (100 μg/kg) and D-GalN (800 mg/kg) through the tail vein to induce the model. One hour after the injection of LPS/D-GalN, the model animals were injected with either 5 mg/kg CAT-PEG or equal volume of 1× PBS through the tail-vein. Healthy mice without any treatment were used as control. The survival rates of the mice were monitored for 12 h after CAT-PEG injection.

### Blood Routine and Blood Chemistry Tests

For the blood routine and blood chemistry tests, the blood samples were collected 6 h after the injection of CAT-PEG or PBS to the model mice. For the blood routine examination, 100 µl of blood samples were taken from the orbital venous plexus of the anesthetized mice and placed in an anticoagulant tube. Blood routine examination was then performed using the whole blood by a routine blood test instrument. For the blood chemistry assay, 300–500 µl of blood was collected from the orbital venous cluster of the anesthetized mice and placed in a centrifuge tube at room temperature for 1 h for a clot to form. The serum was separated from the whole blood by centrifugation at 4,000 *g* for 10 min. ALT and AST activities were measured using ALT Activity Assay Kit and AST Activity Assay Kit, respectively, according to the manufacturer’s instructions. TNF-α and IL-6 levels were profiled in the blood of treated animals using murine TNF-α- and IL-6-specific ELISA assays according to the manufacturer’s instructions.

### Histologic Analysis

Six hours after the injection of CAT-PEG or PBS to the model mice, the mice were anesthetized by isoflurane and perfused with 50 ml of cold 1xPBS through the vascular system. Afterwards, the heart, liver, spleen, lung, and kidney tissues were dissected and fixed in 4% paraformaldehyde; and subsequently embedded in paraffin for histologic analysis. Sections (5 μm) were cut and dried, deparaffinized, and rehydrated. Then, the sections were stained with hematoxylin-eosin (H and E) using a standard protocol and analyzed by light microscopy. Terminal deoxynucleotidyl transferase dUTP nick-end labeling (TUNEL) assay was performed using a TUNEL Apoptosis Detection Kit according to the manufacturer’s protocol. The nuclei were stained with DAPI and further imaged by a Confocal Laser Scanning Microscope.

### Statistical Analysis

All results are presented as the mean ± standard error of the mean (s.e.m.) as indicated. Paired t-tests and one-way ANOVA were used for multiple comparisons (when more than two groups were compared). All statistical analyses were conducted with Prism Software (Prism 8.0).

## Result

### Characterization of Catalase and Catalase-PEG

**FIGURE 1:**
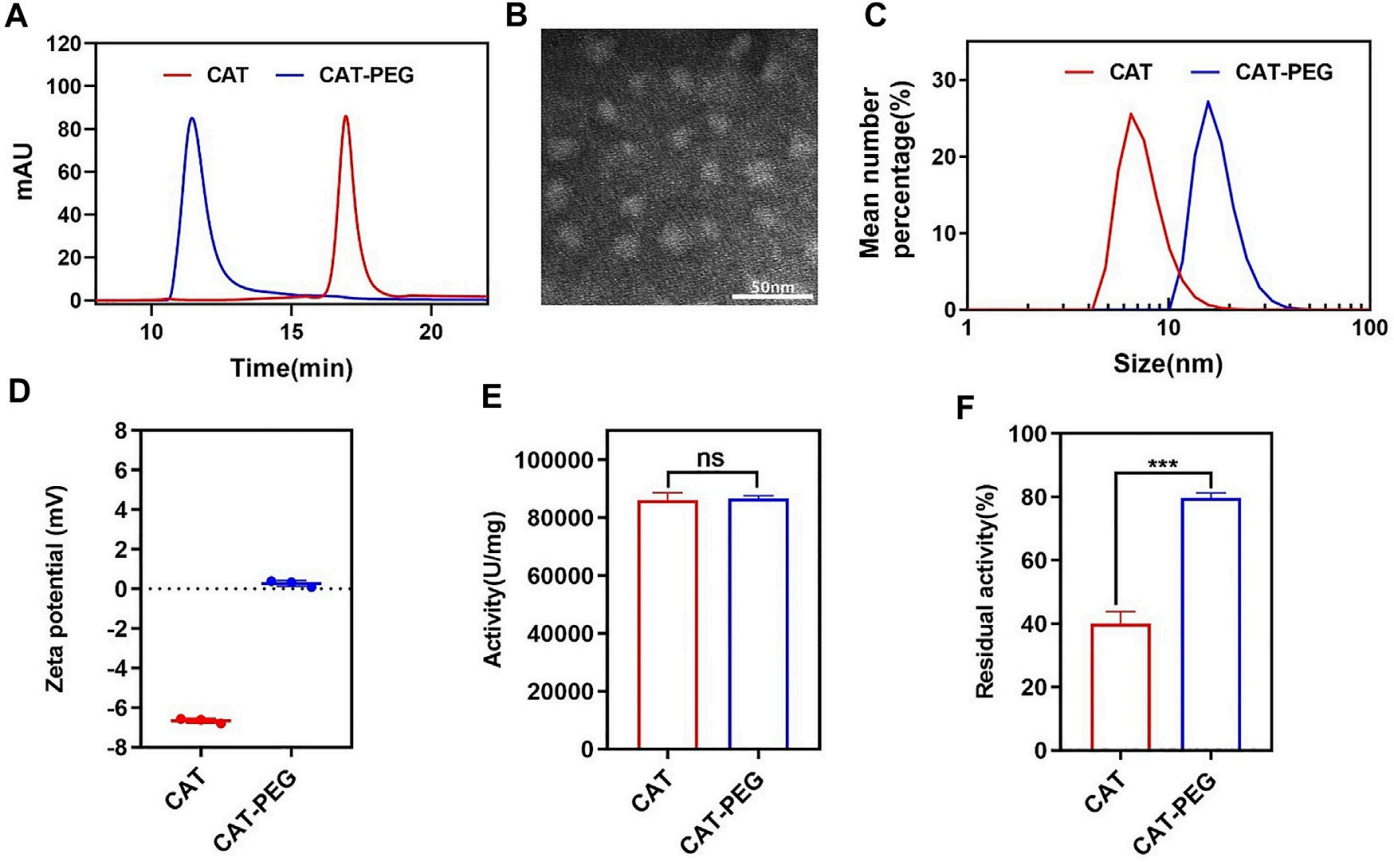
Characteristics of CAT and CAT-PEG. **(A)** HPLC of CAT and CAT-PEG; **(B)** TEM image of CAT-PEG; **(C)** DLS and **(D)** zeta potential of CAT and CAT-PEG; **(E)** The enzyme activity and **(F)** proteolytic stability of CAT and CAT-PEG. *n* = 3. Data represent means ± s.e.m. ***p＜0.001. ns means not significant.

Figure 1A shows high pressure liquid chromatography (HPLC) of CAT and CAT-PEG, denoted respectively as CAT and CAT-PEG hereinafter. Compared with CAT, CAT-PEG shows a shorter retention time indicating successful conjugation of CAT with PEG (Figure 1A). Sodium dodecyl sulphate-polyacrylamide gel electrophoresis (SDS-PAGE) revealed CAT-PEG mainly remained in the upper end due to the conjugation with PEG (Supplementary Figure S1). Transmission electron microscopic (TEM) image confirmed CAT-PEG has an average size of 15 nm (Figure 1B). Consistently, dynamic light scattering (DSL) suggests CAT-PEG displays a size distribution centered at 17 nm and zeta potential near neutral, in comparison with CAT with a size centered at 10 nm and a negative zeta potential of 6.5 mV (Figures 1C,D). The changes of CAT in size and zeta potential after PEG conjugation are consistent with previous studies, which can be attributed to the formation of the PEG layer around the CAT and the shielding effect by such a PEG layer (Harris and Chess, 2003). Compared with CAT, CAT-PEG exhibited a similar enzymatic activity (Figure 1E) and a significantly enhanced enzyme stability (Figure 1F). After incubation in PBS with 1 mg/ml trypsin at 37°C for 4 h, CAT-PEG and CAT retained 79 and 40% of the activity, respectively. This is mainly due to the hydration layer formed by the combination of PEG and the hydrogen bond of water molecules in the solution, which protects the active center of the protein and makes it difficult to be hydrolyzed by protease (Harris and Chess, 2003). Furthermore, after incubation with PBS at 37°C for 120 h, CAT-PEG still retained 90% of its activity (Supplementary Figure S2), which will be beneficial for its *in vivo* use.

### The Ability of Catalase-PEG to Eliminate the Intracellular Reactive Oxygen Species and Prevent Cell Injury

**FIGURE 2:**
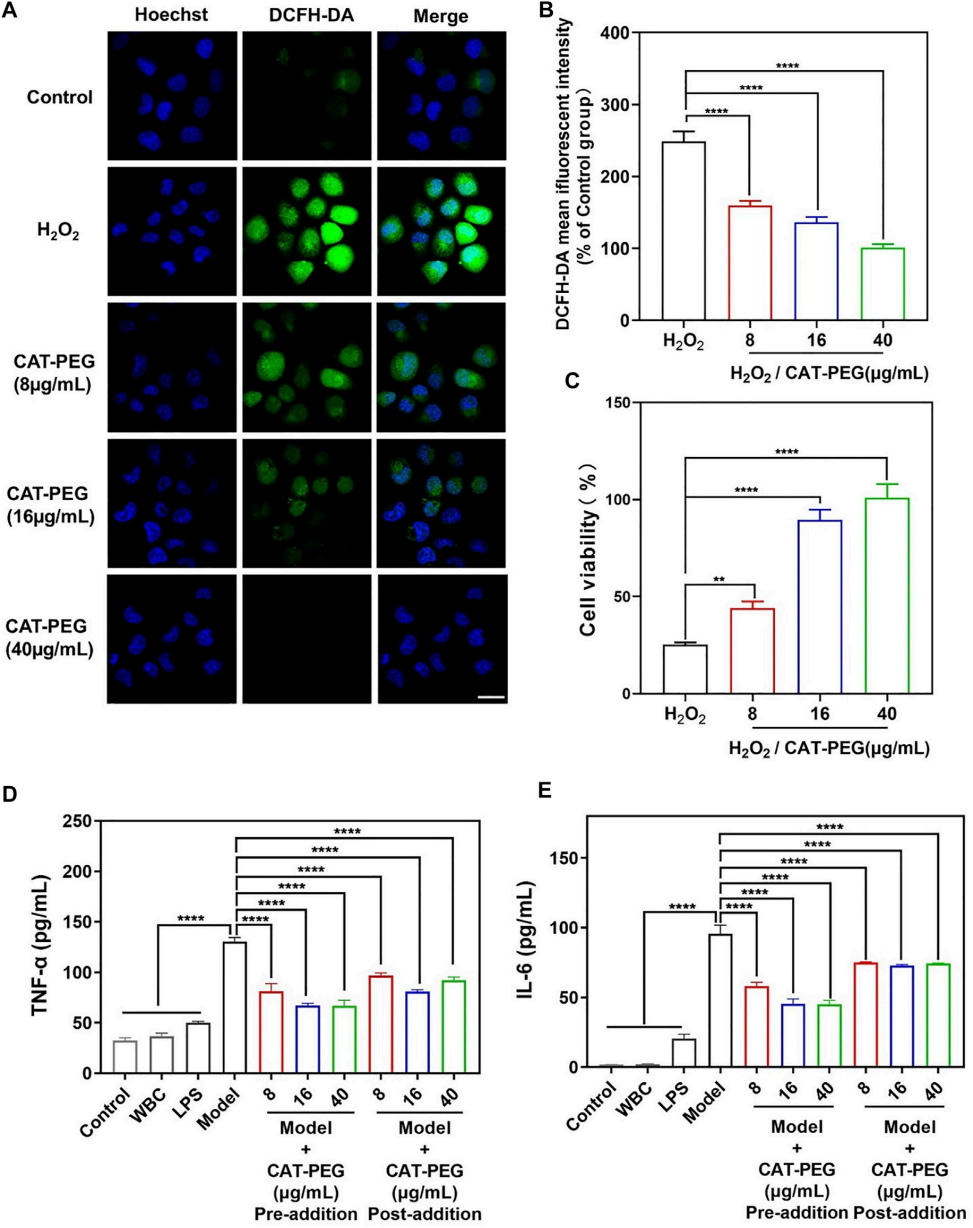
The ability of CAT-PEG to reduce ROS level, prevent cell injury and to regulate the production of inflammatory factors. **(A)** Fluorescence images of HPAEpi cells after incubation with CAT-PEG for 12 h followed by adding 1 mM H_2_O_2_ and 10 μM DCFH-DA and incubating for 30 min (green: ROS, blue: nuclei). Scale bar: 50 μm. **(B)** Relative fluorescent intensity of DCFH-DA in the HPAEpi cells after incubation with CAT-PEG for 12 h followed by adding 1 mM H_2_O_2_ and 10 μM DCFH-DA and incubating for 30 min (% of Control group). **(C)** Cell viability of the HPAEpi cells with 8 μg/ml, 16 μg/ml, 40 μg/ml of CAT-PEG for 12 h, followed by addition of H_2_O_2_ (1 mM) and culturing for 24 h **(D, E)** The concentration of TNF-α (D) and IL-6 (E) in the media of HPAEpi cells cultured with WBC, LPS, and different concentrations of CAT-PEG. In the Pre-addition study, HPAEpi cells were first cultured with different concentrations of CAT-PEG for 12 h, followed by adding LPS (1 μg/ml) and culturing for 24 h. In the Post-addition study, HPAEpi cells were cultured with 1 μg/ml LPS for 24 h, followed by culturing in fresh media containing different concentrations of CAT-PEG for 12 h *n* = 3. Data represent means ± s.e.m., p value: *p＜0.05, **p＜0.01, ***p＜0.001 and ****p＜0.0001. ns means not significant.

We first investigated the cytotoxicity of CAT-PEG by culturing HPAEpi cells with different concentrations of CAT-PEG (Supplementary Figure S3). The cells with CAT-PEG exhibited similar or higher cell viability than the control cells, indicating that CAT-PEG did not show any noticeable cytotoxicity to HPAEpi cells. To investigate the ability of CAT-PEG to eliminate the intracellular ROS, the HPAEpi cells were first incubated with different concentrations of CAT-PEG, followed by exposing the cells to H_2_O_2_, a commonly used intracellular ROS inducer, to induce a cell injury model. The intracellular ROS was subsequently detected with DCFH-DA, a fluorescent probe for ROS. Figure 2A shows the fluorescence images of the HPAEpi cells after different treatments. The cells with H_2_O_2_ show highly intense fluorescence (high level of intracellular ROS), whereas the cells with CAT-PEG show decreased fluorescence signal as the CAT-PEG concentration increased from 8 μg/ml to 40 μg/ml, confirming CAT-PEG can effectively eliminate the intracellular ROS. Further quantitative results showed that the H_2_O_2_-treated cells exhibit 2.5-folds higher fluorescence intensity than those cells with 40 μg/ml of CAT-PEG treatment (Figure 2B). Such an eliminated ROS ability by CAT-PEG can prevent the cells from oxidative injury. As shown in Figure 2C, the cells without CAT-PEG treatment show only a viability of 25%, while the cells with the treatment of 8 μg/ml, 16 μg/ml and 40 μg/ml of CAT-PEG retain 43, 89 and 100% of the cell viability, respectively. These results suggest that CAT-PEG can reduce the intracellular ROS level of the HPAEpi cells and prevent the cells from oxidative injury.

### The Ability of Catalase-PEG to Regulate the Production of Inflammatory Factors

Sepsis is a systemic inflammatory response syndrome caused by infection and mediated by immune cells (Matsuda et al., 2012). In the early stage of inflammation, release of pro-inflammatory factors such as TNF-α and IL-6 stimulates the endogenous immune response. However, in patients with sepsis, the uncontrolled release of such cytokines may cause a series of pathological damages (Cai et al., 2010). In addition, such pro-inflammatory cytokines may also activate the blood coagulation system and upregulate the expression of inflammatory mediators, leading to the occurrence of life-threatening syndrome (Kast, 2000). In this context, regulating the production of cytokines is essential to restore a homeostasis, and TNF-α antagonist has been suggested for the alleviation of hyperinflammation in severe cases (Marchesoni et al., 2009).

The ability of CAT-PEG to regulate the production of cytokines was studied using human leukocytes (white blood cells, WBC) and HPAEpi cells. To test the ability of CAT-PEG to regulate the production of cytokines, leukocytes and HPAEpi cells were cultured with lipopolysaccharides (LPS, a bacterial endotoxin that activates leukocytes) with and without CAT-PEG. The cells without CAT-PEG treatment showed significantly increased secretion of both TNF-α and IL-6. The cells with CAT-PEG showed dramatically reduced levels of TNF-α and IL-6 when the leukocytes were pre-cultured with CAT-PEG followed by adding LPS (Pre-addition) or pre-cultured with LPS followed by addition of CAT-PEG (Post-addition) (Figures 2D,E). This study suggests that CAT-PEG can downregulate the production of TNF-α and IL-6 by the activated leukocytes.

**FIGURE 3:**
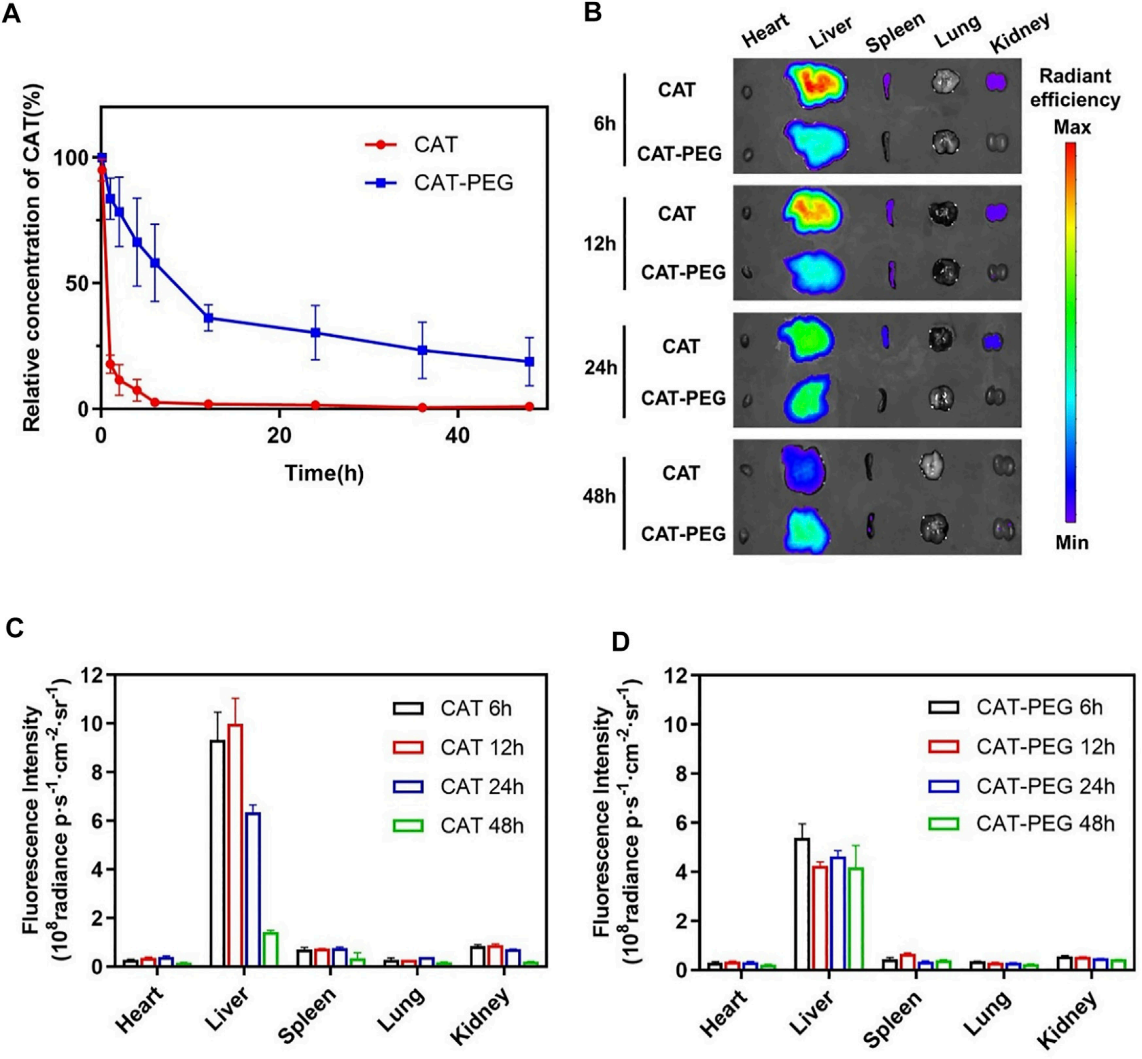
Pharmacokinetics and biodistribution of CAT and CAT-PEG. **(A)** Pharmacokinetics of CAT and CAT-PEG after intravenous administration of 5 mg/kg^−1^ CAT or CAT-PEG. **(B)** Fluorescence imaging of the major organs after intravenous administration of 5 mg/kg^−1^ sulfo-Cy7-labeled CAT or CAT-PEG. **(C, D)** Quantitative analysis of the accumulation of CAT (C) and CAT-PEG (D) in the organs. *n* = 3. Data represent means ± s.e.m. Pharmacokinetics and biodistribution of CAT-PEG in mice.

For therapeutic use, we first investigated the pharmacokinetics and biodistribution of CAT-PEG in mice using near-infrared fluorescence imaging. Two groups of healthy BALB/c mice were intravenously injected with the same amount of CAT or CAT-PEG. Figure 3A shows the plasma concentrations of CAT and CAT-PEG, where CAT-PEG exhibits a much longer half-life (5.30 h) than CAT (0.36 h), an approximate improvement of 15 folds (Supplementary Table S1). To further investigate the biodistribution of CAT-PEG, same amount of CAT and CAT-PEG was fluorescently labeled with sulfo-Cy7 and intravenously injected to two groups of healthy BALB/c mice respectively. Figure 3B shows the fluorescence imaging of the major organs, including heart, liver, spleen, lung, and kidney, of which the average radiation intensity is shown in Figures 3C,D. Both CAT and CAT-PEG were mainly accumulated in the reticuloendothelial system organs (liver and spleen), of which fluorescence intensity decreased with time. Compared with CAT, CAT-PEG showed significantly lower accumulation in the liver, spleen, and kidney, which is consistent with the ability of PEGylation to prolong the circulation time of a conjugated protein.

### Therapeutic Effect of Catalase-PEG in Mice With Sepsis

**FIGURE 4:**
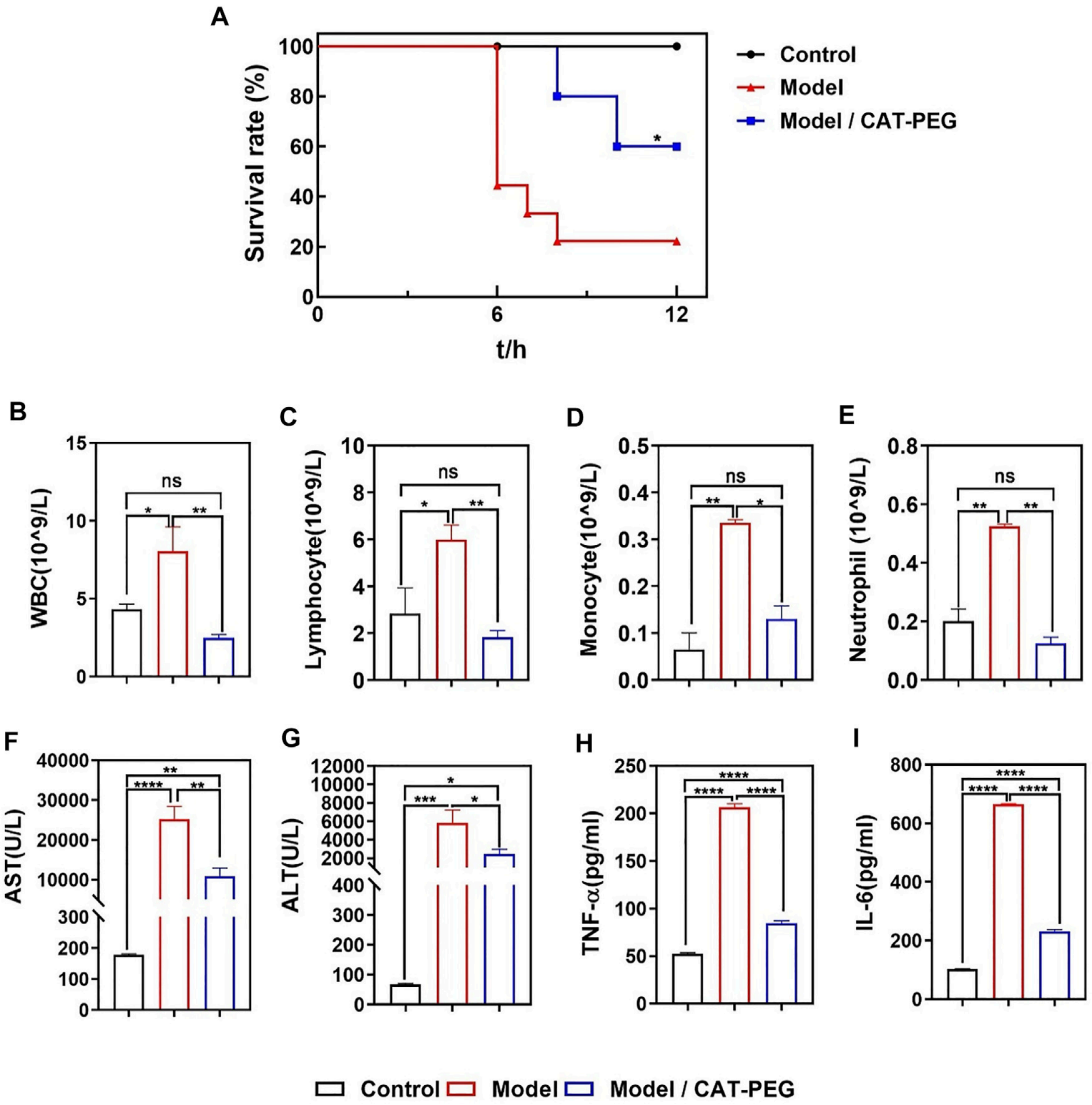
Effectiveness of CAT-PEG treatment in mice with induced sepsis. **(A)** Survival curve of. the healthy mice (Control group), the mice with induced sepsis (Model group) and the model mice with CAT-PEG treatment (Model + CAT-PEG group). The survival of mice was monitored for 12 h n = 9. *p＜0.05 (Log-rank (Mantel-Cox) test). **(B–E)** counts of white blood cells (WBC), lymphocytes, monocytes, and neutrophils, **(F)** aspartate aminotransferase (AST), **(G)** alanine aminotransferase (ALT), **(H)** TNF-α, **(I)** IL-6 levels of the control group, model group, and treatment group. *n* = 3. Data represent means ± s.e.m. p value: *p＜0.05, **p＜0.01, ***p＜0.001 and ****p＜ 0.0001. ns means not significant.

The sepsis model was established by tail-vein injection of LPS (100 μg/kg) and D-galactosamine (D-GalN, 800 mg/kg) to the mice. To evaluate the therapeutic effect of CAT-PEG in mice with sepsis, the model animals were injected with either 5 mg/kg CAT-PEG or equal volume of 1× PBS through the tail-vein 1 h after the injection of LPS/D-GalN. Healthy mice without any treatment were used as control. As shown in Figure 4A, 60% of the mice died within 6 h in the model group, with a survival rate of 20% after 12 h. In the CAT-PEG treatment group, death of the mice occurred after 8 h, with a significantly higher survival rate of 60% after 12 h. Figures 4B–E further compares the counts of the inflammatory cells in the blood samples of the mice from the model group and the CAT-PEG treatment group. Compared with the control group, the model group showed significantly increased counts of the white blood cells (WBC), lymphocytes, monocytes, and neutrophils. In contrast, the model mice showed significantly lowered counts of these inflammatory cells after CAT-PEG treatment, which are similar to those of the control group. These results suggest that CAT-PEG treatment significantly reduced the inflammation syndrome of the model animals. In addition, the liver is one of the organs most affected by sepsis (Yan et al., 2014). The model group showed significantly elevated levels of serum aspartate aminotransferase (AST) and alanine aminotransferase (ALT), which were also reduced significantly in the treatment group (Figures 4F,G). Similarly, compared with the control group, the model group also showed significantly elevated levels of TNF-α and IL-6, which were significantly reduced after the CAT-PEG treatment (Figures 4H,I).

### Histologic Analysis

**FIGURE 5:**
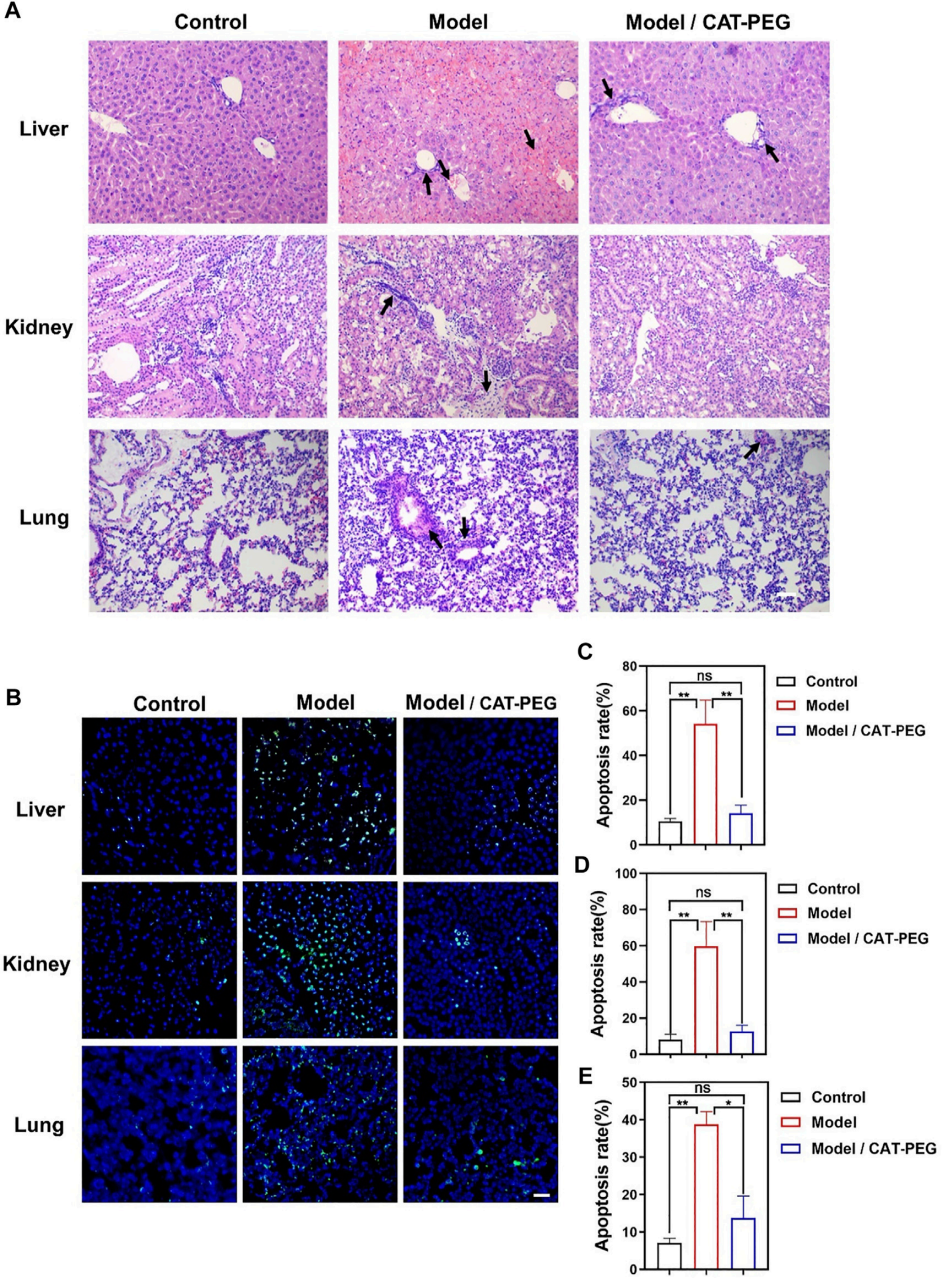
Histologic analysis of the control, model, and treatment group. **(A)** Representative H and E staining sections of the major organs (Original Magnification ×200). **(B)** Terminal deoxynucleotidyl transferase dUTP nick-end labeling (TUNEL) staining of the liver, kidney, and lung sections. Scale bar: 50 μm. The nucleus was stained with DAPI (blue). Apoptotic cells were stained showing green color. **(C–E)** The percentage of TUNEL positive cells, which was counted by Leica TCS SP8 Confocal laser scanning microscope for each section and counted at least three viewing fields in each experiment. The tissue from top to **bottom:** liver, kidney, and lung. *n* = 3. Data represent means ± s.e.m. *p＜0.05 and **p＜0.01. ns means not significant.

Figure 5A shows the hematoxylin-eosin (H and E) staining of the liver, lung and kidney in the model and treatment groups. In the model group, the liver cells were swollen and deformed, the liver cords were not arranged neatly, and liver sinusoid congestion and necrosis were serious. For lung tissue, the wall thickness of the alveoli was significantly thicker than that of the control group, and the alveoli were partially congested, accompanied by a large amount of inflammatory cell infiltration. For the kidney, the structure of the kidney tissue was disorderly arranged, the extracellular matrix increased more than normal, a large number of inflammatory cells infiltrated, and the necrosis of renal tubular epithelial cells was serious. On the contrary, the above-mentioned pathological changes were significantly reduced in the treatment group. Similarly, the treatment group also showed reduced damage in the heart and spleen (Supplementary Figure S4). Specifically, compared with the control group, the arrangement of myocardial fibers was disordered, myocardial cells were swollen, and the interstitium of the myocardial cells widened, accompanied by inflammatory cytokine infiltration in the model group. For the spleen, the proportion of red and white pulp was imbalanced with irregular distribution, the spleen was severely congested, and the cell density of the lymphatic sheath around the arteries was significantly reduced. Figure 5B shows the terminal deoxynucleotidyl transferase dUTP nick-end labeling (TUNEL) staining of the liver, kidney, and lung sections. Compared with the control group, the apoptotic cells in the liver, kidney, and lung of the model groups increase significantly. In contrast, the apoptotic cells are significantly reduced in after the CAT-PEG treatment. Further quantitative analysis results show that the CAT-PEG treatment resulted in 4-fold, 6-fold and 3-fold lowered apoptosis rate in the liver, kidney, and lung than the model group, respectively (Figures 5C–E).

## Discussion

Sepsis involves complex network effects of multiple systems, including inflammation, immune dysfunction, coagulation dysfunction, endothelial injury, multiple organ injury, and shock, etc. We have shown that CAT-PEG can effectively downregulate the production of TNF-α and IL-6 by activated leukocytes, suppress the level of AST, ALT, TNF-α, and IL-6 in the mice with induced sepsis, leading to significantly reduced mortality rate. TNF-α is a pleiotropic cytokine that mediates the inflammatory cascade though inducing cytokines such as IL-6 (Zhang et al., 2010), as well as cell apoptosis in the early stage through a series of signal transmissions (Kaplowitz, 2000). Therefore, TNF-α and IL-6 are related to a wide range of inflammatory or autoimmune diseases. In this study, administration of LPS/D-GalN significantly increased the levels of plasma TNF-α and IL-6, which was restored after CAT-PEG treatment, leading to reduced apoptosis of the liver, lung, kidney and other organs.

In addition to being a weapon against pathogens, ROS is also involved in many physiological processes, serving as both an inflammatory signaling molecule and an inflammatory mediator (Mittal et al., 2014). Long-term or chronic ROS molecules are considered to be the core of the development of inflammatory diseases (Pendyala and Natarajan, 2010). As a representative of ROS, increasing number of studies have shown that H_2_O_2_ plays an important role in the physiology of cells and organs (Rhee, 2006; Sies, 2014). For example, H_2_O_2_ plays an irreplaceable role as a “messenger” and “guide” during an inflammatory response (Wittmann et al., 2012). The presence of H_2_O_2_ is necessary for the release of pro-inflammatory cytokines and regulate an appropriate immune response (Nathan and Cunningham-Bussel, 2013), whereas excessive production of ROS including H_2_O_2_ could cause immunopathogenesis. We speculate that CAT-PEG can also regulate the production of cytokines, restoring immune balance by removing excess ROS produced.

In summary, we have demonstrated the PEGylated catalase can effectively regulate the production of cytokines by leukocytes, suppress the elevated level of AST, ALT, TNF-α, and IL-6 and mitigate the damage to the liver, kidney, lung function and other organs in mice with induced sepsis. These factors collectively contributed to a dramatic increase of survival rate. These findings suggest that CAT-PEG may provide an efficient therapeutic solution to sepsis, as well as other hyperinflammation-related diseases.

## Data Availability

The original contributions presented in the study are included in the article/Supplementary Material, further inquiries can be directed to the corresponding authors.
