# Supplementary material for: An Antioxidant Enzyme Therapeutic for Sepsis
Source: Front Bioeng Biotechnol. 2021 Nov 23;9:800684. doi: 10.3389/fbioe.2021.800684 (PMC8650590; doi:10.3389/fbioe.2021.800684)
Supplement: Supplementary file 1 [file DataSheet1.DOCX]

Supplementary Material


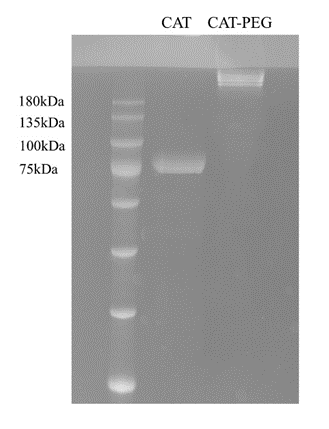


**Supplementary Figure 1.** SDS-PAGE of the CAT and CAT-PEG.


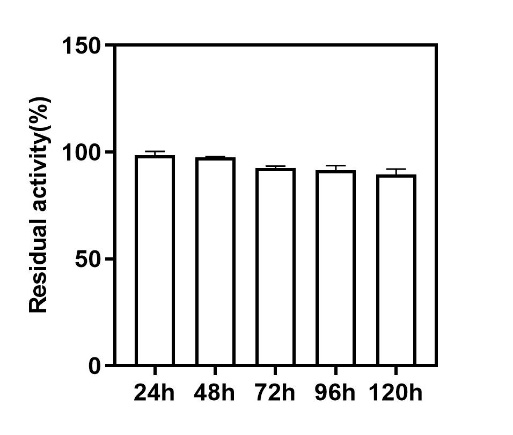


**Supplementary Figure 2.** The stability of CAT-PEG. Residual activity after CAT-PEG diluted with PBS to a final concentration of 0.1 mg/mL and incubated at 37°C for 24h, 48h, 72h, 96h and120h.


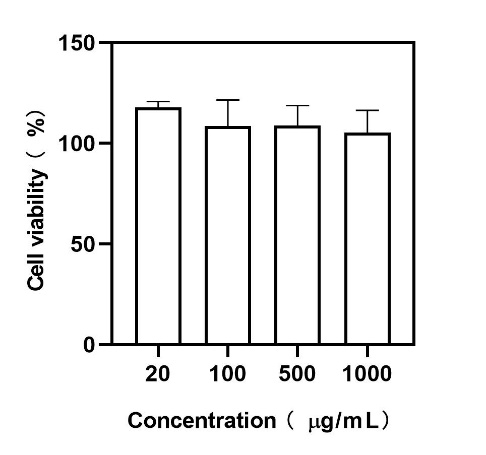


**Supplementary Figure 3.** Cytotoxicity of CAT-PEG. Cell viability of HPAEpiC in the presence of different concentrations of CAT-PEG for 24h.


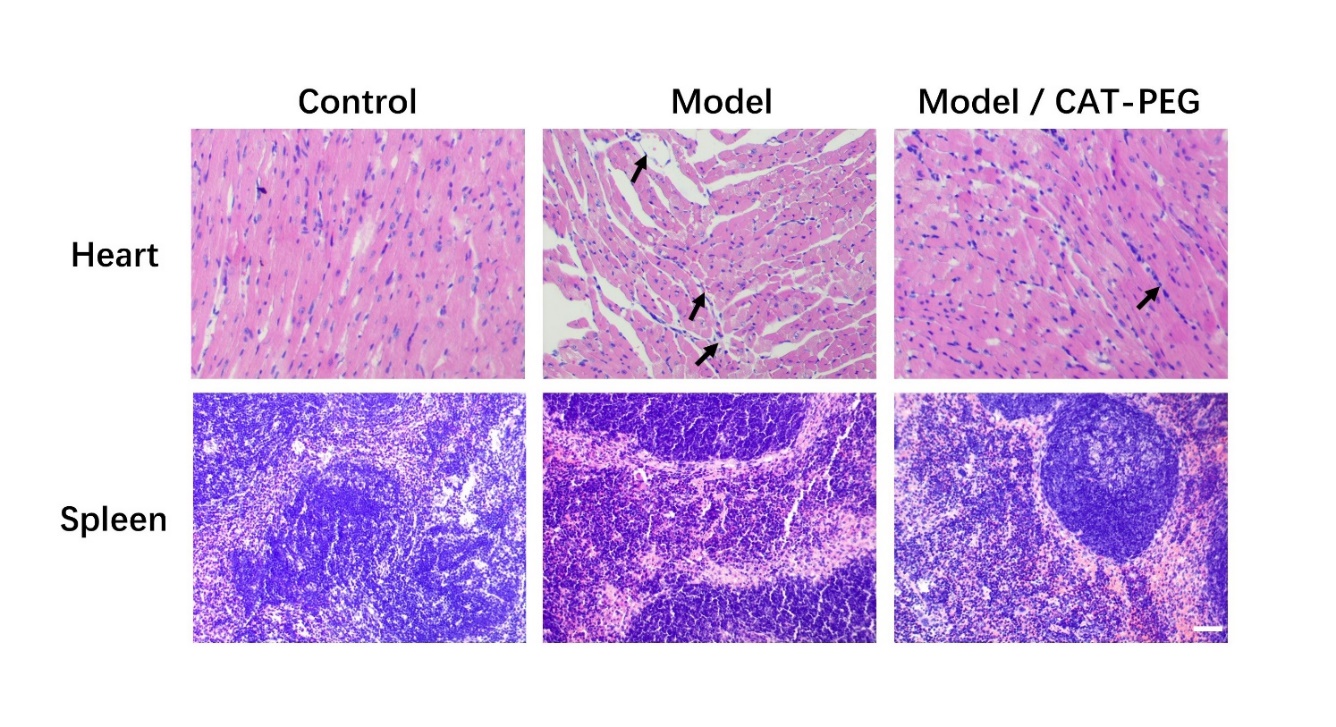


**Supplementary Figure 4.** H&E staining sections of heart and spleen in BALB/c mice 12 h after CAT-PEG (5mg/kg) treatment. Scale bar: 50 µm.

**Table S1 | Pharmacokinetics parameters of native CAT and CAT-PEG.** The analysis of the PK data was achieved by fitting the data using a one-phase exponential decay model. All data analysis was achieved using Graphpad Prism 8.0.

| Parameters | CAT | CAT-PEG |
| --- | --- | --- |
| k (h^-1^) | 1.931 | 0.1309 |
| t_1/2_ (h) | 0.3590 | 5.296 |
| τ (h) | 0.5180 | 7.641 |
| AUC(U/mL*min) | 149.9 | 1691 |
